# Tissue Culture of Isolated Human Pancreatic Islets Infected With Different Strains of Coxsackievirus B4: Assessment of Virus Replication and Effects on Islet Morphology and Insulin Release

**DOI:** 10.1155/EDR.2000.165

**Published:** 2000

**Authors:** Gun Frisk, Hans Diderholm

**Affiliations:** 1 Department of Women's and Children's Health/Paediatrics Akademiska Hospital Uppsala S-751 23 Sweden; 2 Department of Medical Sciences/Infectious Diseases and Clinical Virology Uppsala Sweden

## Abstract

The aim was to study whether different strains of Coxsackievirus B4 (CBV-4) are able to infect human pancreatic islet cells * in vitro* and cause morphological and functional damages. Isolated islets maintained in tissue culture were infected with five well- characterised strains of CBV-4. Aliquots of the culture medium were analysed with regard to virus replication and insulin content. Infected and uninfected islets were examined by light microscopy to determine the degree of virus-induced cytopathic effect (CPE). The results showed that the islet cells were susceptible to infection by all the strains of CBV-4 although the outcome of the infection differed. The virus titres obtained at 48 and 72 hours post infection differed significantly between all the CBV-4 strains (*p* < 0.001), indicating different ability to replicate in islet cells. Pronounced to weak CPE, which was partly due to the origin (donor) of the islets, was induced by four of the five CBV-4 strains. One strain (VD2921) replicated without causing CPE despite high virus titres. One (V89-4557) of the CBV-4 strains always revealed pronounced CPE. Infection by this strain also caused functional impairment that significantly affected insulin response to high glucose at 48 hours post infection (*p* < 0.001). Replication of another CBV-4 strain (JVB) in the islet cells significantly increased the release of insulin compared to non-infected control cells (*p* < 0.001) indicating damage of the β-cells leading to leakage of insulin.


*Int. Jnl. Experimental Diab. Res.*, Vol. 1, pp. 165–175
Reprints available directly from the publisher
Photocopying permitted by license only

© 2000 OPA (Overseas Publishers Association) N.V.
Published by license under
the Harwood Academic Publishers imprint,
part of The Gordon and Breach Publishing Group.
Printed in Malaysia.

### Tissue Culture of Isolated Human Pancreatic Islets Infected with Different Strains of Coxsackievirus B4: Assessment of Virus Replication and Effects on Islet Morphology and Insulin Release

GUN FRISK[a,*] and HANS DIDERHOLM[b]

[a]*Department of Women's and Children's Health/Paediatrics;* [b]*Department of Medical Sciences/Infectious Diseases and Clinical Virology, Uppsala, Sweden*

*(Received in final form 3 March 2000)*

**The aim was to study whether different strains of Coxsackievirus B4 (CBV-4) are able to infect human pancreatic islet cells *in vitro* and cause morphological and functional damages. Isolated islets maintained in tissue culture were infected with five well- characterised strains of CBV-4. Aliquots of the culture medium were analysed with regard to virus replication and insulin content. Infected and uninfected islets were examined by light microscopy to determine the degree of virus-induced cytopathic effect (CPE). The results showed that the islet cells were susceptible to infection by all the strains of CBV-4 although the outcome of the infection differed. The virus titres obtained at 48 and 72 hours post infection differed significantly between all the CBV-4 strains ($p<0.001$), indicating different ability to replicate in islet cells. Pronounced to weak CPE, which was partly due to the origin (donor) of the islets, was induced by four of the five CBV-4 strains. One strain (VD2921) replicated without causing CPE despite high virus titres. One (V89-4557) of the CBV-4 strains always revealed pronounced CPE. Infection by this strain also caused functional impairment that significantly affected insulin response to high glucose at 48 hours post infection ($p<0.001$). Replication of another CBV-4 strain (JVB) in the islet cells significantly increased the release of insulin compared to non-infected control cells ($p<0.001$) indicating damage of the β-cells leading to leakage of insulin.**

*Keywords*: Human ß-cells; Pancreatic islets; Enterovirus; Coxsackievirus; Diabetes mellitus; Insulin release

### INTRODUCTION

There is much evidence to suggest that viral infections are of etiologic significance for the development of insulin-dependent diabetes mellitus (IDDM).[1,2] The congenital rubella syndrome is a well-documented cause of IDDM,[3,4]

*Corresponding author. Uppsala University, Department of Women's and Children's Health/Paediatrics, Akademiska Hospital, S-751 23 Uppsala, Sweden. e-mail: Gun.Frisk@pediatrik.uu.se and epidemiological studies have linked outbreaks of mumps with clusters of IDDM cases.[4] There is accumulating evidence that also enteroviruses, especially Coxsackie B viruses (CBV), are of etiologic significance in this context.[2,5] Higher frequencies of neutralising antibodies against CBV have been seen in IDDM patients compared with healthy controls. CBV–IgM, CBV-derived RNA, and IgM antibodies against other enteroviruses were found in higher frequencies in newly diagnosed patients than in controls.[6–11] More direct evidence has been obtained from isolation of CBV, capable of causing diabetes in mice, from the pancreas of a few patients with acute onset IDDM.[12,13]

It has been suggested that induction of IDDM by CBV may reflect persistent viral infections, which initiate autoimmune mechanisms directed against the pancreatic Beta-cells.[14] Autoimmunity against these cells may result from viral antigens sharing conformational or structural features with Beta-cell autoantigens, which leads to a breakdown of immunologic tolerance. Molecular similarities between the non-structural P2C protein of CBV and islet GAD65 (glutamic acid decarboxylase) have been demonstrated.[15,16] Alternatively, bacterial toxins or human endogenous retroviruses may act as superantigens that induce the formation of autoreactive T-cell clones directed against the Beta-cells.[17] This view, however, has been strongly disputed in more recent studies.[18] There is also the recently proposed possibility that CBV infection of islet cells causes the release of sequestered islet antigens resulting in stimulation of resting autoreactive T-cells and inflammatory Beta-cell damage.[19]

Although CBV may thus be an exogenous factor of importance for the development of IDDM, there is still a considerable lack of knowledge as to the mechanisms involved. Animal models are not always representative of the clinical situation as exemplified in previous studies indicating that human ß-cells are much more resistant to ß-cytotoxins and cytokine-induced cell damage than rodent ß-cells [20] Experiments at the cellular levels have been hampered by a limited access to isolated human islets. It has been shown that the tropism of different strains of CBV-4 differs, *e.g.*, not all strains can bind to and replicate in ß-cells from mice.[21,22] The present study is an attempt to test the hypothesis that some strains of CBV-4 are not able to infect human islet cells causing morphological and functional damage to these cells. For this purpose we have studied the interactions between five well-defined strains of CBV-4 and isolated human islets *in vitro* with respect to virus replication, insulin release in response to glucose and morphological signs of cellular destruction.

### MATERIALS AND METHODS

### Preparation and Culture of Isolated Human Islets

Human pancreata were excised from 32 heart-beating organ donors aged 41 ± 2 years (range 15–63 years; 18 men, 14 women) and transported to the Central Unit of Beta-Cell Transplant, Brussels, where islet isolation was performed as previously described.[23] Samples of the isolated islets were examined by electron microscopy and by light microscopy after immunocytochemical staining for insulin and glucagon. There were less than 7% dead cells (6.2 ± 0.7%) and virtually no exocrine cells. The prevalence of insulin positive cells in the isolated islets was 55 ± 2% and that of glucagon positive cells 15 ± 3%. The insulin content was 1.51 ± 0.14 μg/μg islet DNA. The islets were cultured in Brussels for 13 ± 1 days (range 2 – 32) before being sent by air to Uppsala, Sweden. After arrival in Uppsala the islets were immediately suspended in groups of 50 – 150 islets/dish in 5 mL RPMI 1640 containing 5.5 mM glucose and supplemented with 10% foetal calf serum, benzyl penicillin (100 U/mL) and streptomycin (0.1 mg/mL).[24]

### Viruses

Five strains of CBV serotype 4 (CBV-4) were used. Three of them, namely V89-4557, VD2921 and V345, were plaque-purified after isolation from patients suffering from aseptic meningitis. The fourth strain, CBV-4-E2, was kindly provided by Dr. Tapani Hovi, National Public Health Institute, Helsinki, Finland, and has previously been found to be diabetogenic in mice.[25] This strain was derived from the boy Edvards. The fifth CBV-4 strain, JVB, generally considered to be the prototype of CBV-4, was obtained from ATCC (American Type Culture Collection). Neutralisation tests were used to confirm the serotype of the viruses before the inoculation of the islets cells. Poliovirus serotype 1 (PV-1), strain Sabin, was used for comparative purposes and was also obtained from ATCC.

### Virus Replication and Cytopathic Effect (CPE)

Studies of replication of the CBV-4 and PV-1 strains in isolated islets were performed either without changes of culture medium during the first seven days, or before changes of culture medium at 24 h intervals for measurements of insulin release (see below). Islets were inoculated at 37°C with $10^3-10^4$ tissue culture infectious doses 50 ($TCID_{50}$) per dish. In the studies without medium changes each virus was tested for replication in three to five separate islet preparations. When islets were inoculated with the strains V89-4557, VD2921, V345 or PV-1, the numbers of islets were 60–100/well (mean 80), while they were 20–35/well (mean 25) in the studies with CBV-4-E2 and JBV. Each islet consisted of about 1,000 cells. To be able to show replication in this very small number of cells (20–35 islets) we included a negative control consisting of the virus and the culture medium without any cells. After allowing the virus to attach for 30–60 min at 37°C, the islets were resuspended in fresh RPMI 1640 supplemented as described above. The islets were examined each day in a microscope for virus-induced morphological changes, *i.e.*, cytopathic effect (CPE), which was ranked from 0 to 4 (0, no CPE compared to the uninfected control; 1 + weak changes: 5–25% of the islets were disrupted; 2 +, weak to moderate changes: 25–50% of the islet was disrupted; 3 +, severe disruption of the islets: 50–90% of the islet was disrupted; 4 +, the islets were totally disrupted resulting in a cell suspension). Infected islets showing a degree of CPE of 3–4 were stained with 0.2% trypanblue to estimate the number of dead cells. In the study of insulin response, where each strain was tested in 5–10 separate islet preparations, samples of culture media were withdrawn every day for assays of virus. This was also done from islet cultures in which the medium was changed at 24 h intervals. The assays of virus replication were performed by $TCID_{50}$ titrations on Green Monkey Kidney (GMK) cells.

### Measurements of Insulin Release

Groups of 20–100 isolated islets were suspended in 5 mL tissue culture medium and infected with each of the CBV-4 strains or with PV-1 as described above. For each donor a group of non-infected islets served as control. After the initial 24 h culture period the medium was changed and the islets maintained for another 24 h at 5.5 mM glucose. Aliquots of the culture medium were removed and frozen at −20°C for later insulin assays. The culture medium was changed and fresh culture medium added so that wells containing infected or control islets were maintained at either 5.5 or 16.5 mM glucose for the following 24 h, new aliquots were taken for insulin assay. After a final change of culture medium all islets were again cultured at 5.5 mM glucose for 24 h and samples taken for insulin assays. They were performed with Pharmacia Insulin RIA 100 Kit (Pharmacia & Upjohn Diagnostics, Uppsala, Sweden) using a human insulin standard. The detection limit for this assay is $< 2\mu U/mL$ and the precision within an assay 5.7% according to the manufacturer.

### Statistical Analyses

Data presented are means ± SEM each islet preparation, *i.e.*, islets obtained from one donor, was considered as one individual observation. All results were based on observations from at least five donors. A *P*-value of $< 0.05$ was considered significant. Rates of insulin release (μU islet/24 h) from infected and control islets including calculated differences and ratios, have been computed using descriptive statistics. Wilcoxson's signed rank test was used to compare virus infected and control islets with respect to value obtained at 5.5 mM glucose at 72 h post infection (pi), values obtained at 16.5 mM after 72 h pi, differences between values obtained at 5.5 and 16.5 mM after 72 h, values at 5.5 mM after 96 h, and differences between values obtained at 16.5 mM after 72 h and those obtained at 5.5 mM after 96 h. The Kruskal–Wallis' test was used to assess whether the six different viruses differed at 16.5 mM after 72 h with respect to: (a) the infected islet value; (b) the difference between the infected and the control islet value. It is of note that the Kruskal–Wallis'test is based on the assumption of independent groups, which is not entirely fulfilled as islets from one and the same donor have been used to test the effects of several virus strains. This deviation was, however, regarded as insignificant for the outcome. The hypothesis of no difference in titre levels between the six viruses was tested using Kruskal–Wallis'test with respect to values obtained at 72 h pi, values obtained at 96 h pi and difference between values obtained at 96 h pi and 72 h pi.

### RESULTS

### Virus Replication in Islet Cells Over Seven Days

As observed in Figure 1A, the titers (means ± SEM) of the CBV-4 strains V89-4557 and VD 2921, and the PV-1 show that these strains replicated well in the islet cells (titre increase $10^2 - 10^5$/0.2 mL/80 islets). Also the strains CBV-4-E2 and JVB replicated in the islet cells, although the titre increases were only $10^{0.5}$/0.2 mL/25 islets. The negative control showed during the same time a $10^2$/0.2 mL decrease in virus titre. The different replication rates observed with the CBV-4-E2 and JBV strains, may partly reflect the lower islet numbers used in these experiments. Interestingly the JVB strain did not replicate in islets obtained from one donor (H1 205), suggesting variability in the susceptibility to the virus. The strain V345 replicated at varying rates in islets obtained from different donors (Fig. 1B). In two experiments there was only a limited production of this strain. The third experiment showed a clear production of virus, although at a slower rate than the other strains.

### Virus Replication in the Experiments for Insulin Release Determinations

Virus replication was also assessed in the insulin release experiments, in which culture medium was changed every 24th h for up to 96 h pi (Tab. I). At 48 h pi the $TCID_{50}$ titres of each of the five CBV-4 strains and the PV-1 strain were consistently increased above those at 2 h pi. After an additional 48 h-period, and another two changes of culture medium, the titres were even higher for the CBV-4 strains V89-4557, VD2921 and V345, and for the PV-1 strain. The

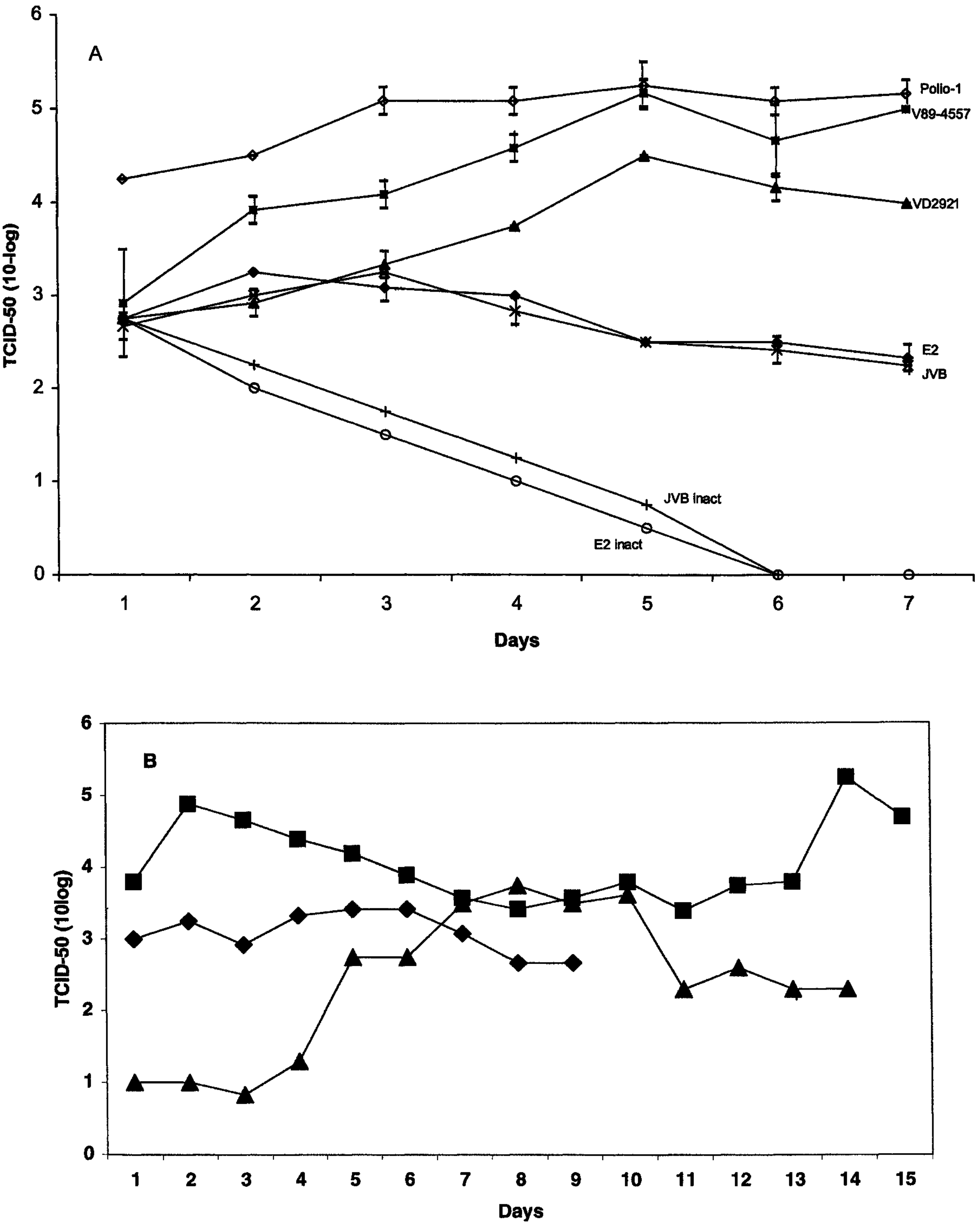

FIGURE 1 A: Virus titres (means ± SEM) in the culture medium of infected human islets. Aliquots of the culture medium were withdrawn every day for seven days and the virus titres were obtained by using the Tissue Culture Infectious Dose$_{50}$ (TCID$_{50}$) titration method. The CBV-4 strains used were V89-4557, VD2921, JVB, CBV-4-E2 and PV-1. As a negative control for the CBV-4 strains CBV-4-E2 and JVB TCID$_{50}$ titrations were performed on aliquots from culture medium from wells not containing any cells. B: Virus titers in the culture medium of three different islets preparations (Δ HI 110, ■ HI 115 and ◆ HI 117) (three diffferent donors) infected with the CBV-4 strain V345. Aliquots of the culture medium were withdrawn every day for seven days and virus titres were obtained by using the TCID$_{50}$ titration method. The infection by this CBV-4 strain did not cause total cytopathic effect (CPE) (not shown).

$TCID_{50}$ titres for the CBV-4 strains JVB and CBV-4-E2 were lower at 96 h pi compared to those obtained at 48 h pi. The Kruskal–Wallis'test showed that the titre levels differed between the strains at 72 h pi ($p < 0.001$), the values obtained at 96 h pi ($p < 0.001$), and the difference between

TABLE I Virus titres (mean ± SEM)obtained in the culture medium of the infected islets by the use of Tissue Culture Infectious $Dose_{50}$ ($TCID_{50}$) titration method in experiments where the culture media were changed at 24, 48 and 72 hours post infection (pi). Four different strains of Coxsackievirus serotype B4 (CBV-4) and one strain of poliovirus serotype 1 (PV-1) were used and virus titers are presented as 10-log. Mean values of the virus induced cytopathic effect (CPE) on the infected islets at the same time points pi as the changes of culture media were performed. For each strain five to ten observations were done

| Virus | | Time post infection | | | | | |
|---|---|---|---|---|---|---|---|
| | | 2 h | | 48 h | | 96 h | |
| strains | *n* | $TCID_{50}titer^{\Psi}$ | CPE | $TCID_{50}$titer | CPE | $TCID_{50}$titer | CPE |
| V89-4557 | 10 | 1.7 ± 1.3 | 0.0 | 2.6 ± 1.5* | 1.1 | 4.0 ± 1.1 | 2.1 |
| VD2921 | 7 | 0.9 ± 0.9 | 0.0 | 1.2 ± 1.0* | 0.0 | 3.3 ± 1.3 | 0.0 |
| V345 | 5 | 0.7 ± 0.1 | 0.0 | 1.4 ± 1.6* | 0.5 | 2.3 ± 1.5 | 1.5 |
| CBV-4-E2 | 6 | 3.2 ± 0.1 | 0.0 | 3.5 ± 0.1* | 0.0 | 2.3 ± 0.1 | 1.0 |
| JVB | 5 | 3.3 ± 0.0 | 0.0 | 3.6 ± 0.2* | 0.3 | 2.9 ± 0.6 | 1.8 |
| PV-1 | 6 | 0.5 ± 0.2 | 0.0 | 2.6 ± 1.7* | 1.2 | 3.3 ± 1.3 | 2.1 |

$^{\Psi}$ = Number of infectious doses per 0.2 mL culture medium.
* = Significant differences between virus titers obtained at 2 h pi and titers obtained at 48 h pi, and, in addition, significant differences between the strains at 48 h pi.

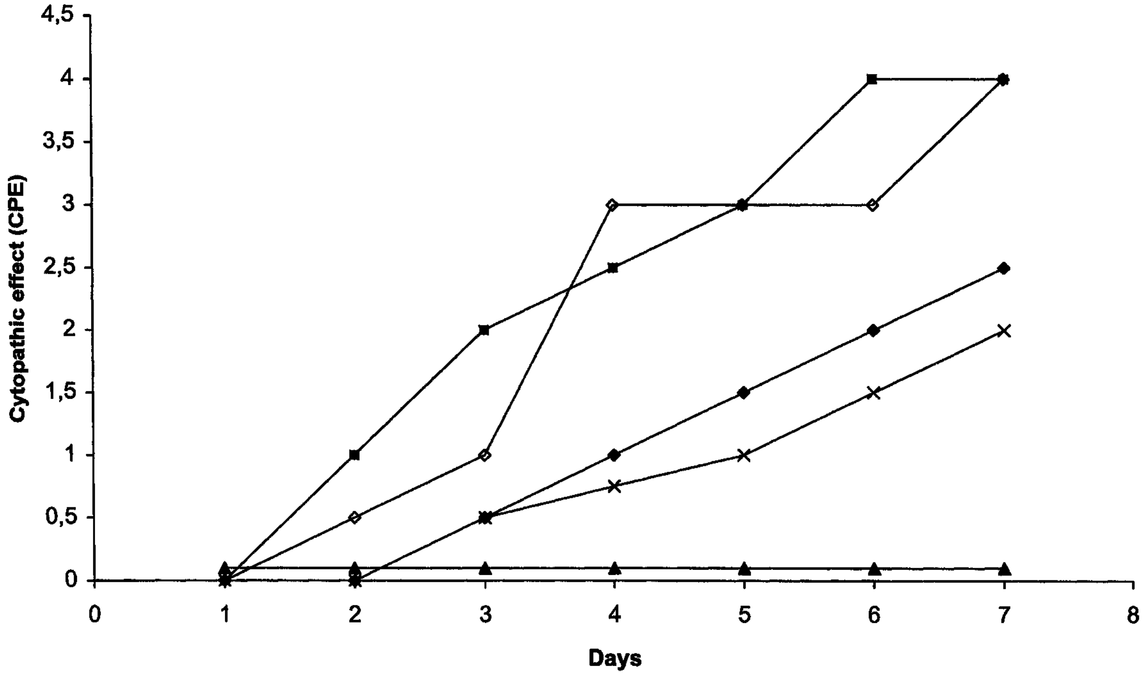

FIGURE 2 Mean values (3–5 observations) of the degree of cytopathic effect (CPE) seen in the infected human islets and islet cells, cultured in RPMI 1640 supplemented with foetal bovine serum, under a light microscope every day for seven days. The degree of CPE was estimated and given a score ranging from 0 to 4 (0 no CPE compared to the uninfected control, 2, 25–50% of the islet was disrupted, 3, 50–90% of the islet was disrupted and 4 the islet were totally disrupted resulting in a cell suspenstion) (■ V89-457, ▲ VD2921, × JVB, ◆ CBV-4-E2, and ◇PV-1) compared to non-infected control islets.

values obtained at 96 h pi and the values obtained at 72 h pi ($p < 0.0001$). All the strains used varied considerably with regard to virus titre and the time of a replication cycle with the origin of the islets cells. This may partly be explained by the different degree of expression of specific virus receptors and to some extent to different conditions of the islets due to different length of the culturing of the islets before infection (6–36 days).

### Morphology of the Islets

The viruses V89-4557 and PV-1 induced strong CPE (4+) leading to total disruption of the islets (resulting in a cell suspension) after seven days (Tab. I, Figs. 2 and 3B). Staining of the cell suspension with 0.2% trypanblue revealed that approximately 70–90% (3+) of the islet cells were dead at seven days pi. The strain VD2921 revealed no CPE and consequently the islets infected with this strain were not stained. Even when islet infected with this strain were followed for up to 26 days they did not show any morphological changes compared with the control islets although the virus continued to replicate in the islet cells (Fig. 3A). The strains V345, CBV-4-E2 and JVB were less cytolytic (2–2.5+) than V89-4557 (4+) and PV-1 (4+) and they did not totally disrupt the islets during an observation period of seven days.

### Insulin Release and Insulin Response to High Glucose

For each virus strain control values were derived from the same islet population (same donor) as the infected islets in order to make possible comparisons between infected and non-infected islets (Tab. II). Despite this, the data on the rates of insulin release in some groups showed rather large variations between individual observations as evidenced by the large standard errors of the means.

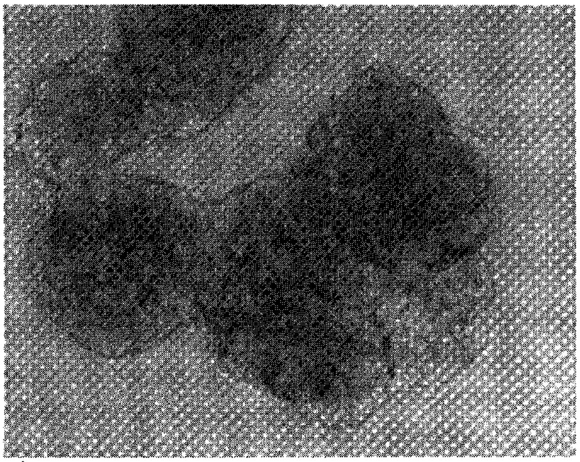

A

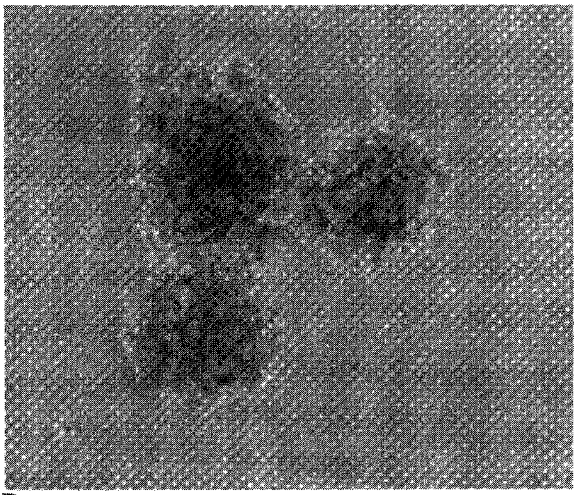

B

FIGURE 3 A: Islets infected with the CBV-4 strain VD2921 and non-infected islets cultured in RPMI 1640 supplemented with 10% foetal bovine serum revealed no cytopathic effect (CPE) at seven days postinfection (pi). In Figure 3A shown islets infected with the CBV-4 strain VD2921 at seven days pi. B: Islets infected with the CBV-4 strain V89-4557 cultured in RMPI 1640 supplemented with 10% foetal bovine serum for five days revealed a strong CPE (3–4). It is possible that the use of 10% FCS might decrease the virus titre but it is appropriate for studying the insulin release in response to high glucose.

The insulin release in the second 24 h-period (48 h pi) was the same from the infected islets as from the control islets. In the third culture period (72 h pi) at 5.5 mM glucose, there was a

TABLE II Insulin release in the culture media from islets infected with different CBV-4 strains and PV-1 and from control islets. Insulin content was measured after 72 hours post infection(pi). The islets have from 48 to 72 hours pi been cultured in media containing 5.5 mM or 16.5 mM glucose. Insulin content of the media was also measured 96 hours pi. The culturing condition from 72 to 96 hours pi was in media containing 5.5 mM glucose

| | | Insulin release*/islet/24hour | | | |
|---|---|---|---|---|---|
| Virus | | 72 hours | | 96 hours | |
| strain | $n$ | 5.5 mM$^{\Psi}$ | 16.5 mM | 5.5 mM$^{\psi}$ | 5.5 mM$^{\delta}$ |
| V89-4557 | 10 | 131 ± 36 | 338 ± 77* | 37 ± 12 | 72 ± 10 |
| Controls | 10 | 148 ± 36 | 602 ± 77 | 69 ± 22 | 123 ± 28 |
| VD2921 | 7 | 110 ± 41 | 406 ± 94 | 109 ± 16 | 96 ± 19 |
| Controls | 7 | 85 ± 32 | 479 ± 72 | 39 ± 16 | 58 ± 9 |
| V345 | 5 | 80 ± 20 | 515 ± 177 | 54 ± 22 | 61 ± 18 |
| Controls | 5 | 63 ± 4 | 601 ± 73 | 36 ± 10 | 61 ± 18 |
| CBV-4-E2 | 6 | 115 ± 80 | 480 ± 325 | 99 ± 28 | 176 ± 166 |
| Controls | 6 | 107 ± 36 | 638 ± 243 | 56 ± 17 | 49 ± 48 |
| JVB | 7 | 333 ± 126 | 626 ± 187 | 239 ± 122* | 299 ± 36* |
| Controls | 7 | 85 ± 30 | 672 ± 195 | 50 ± 13 | 113 ± 36 |
| PV-1 | 6 | 184 ± 76 | 314 ± 71* | 17 ± 11 | 58 ± 31 |
| Controls | 6 | 104 ± 27 | 647 ± 45 | 64 ± 31 | 77 ± 35 |

* = $\mu$U insulin/islet/24 hours.
$^{\Psi}$ = Glucose concentration in the culture media.
$^{\psi}$ = Continuously cultured in 5.5 mM glucose.
$^{\delta}$ = Cultured in 16.5 mM glucose for 24 hours and then in 5.5 mM glucose for additional 24 hours.
* = $P < 0.001$ against the respective controls.

modest, although not significant, increase in the rate of insulin release in islets infected with either the JVB strain of CBV-4 or the PV-1. The simultaneous culture of islets at the 16.5 mM glucose medium showed, however, marked differences between the virus strains with respect to the ability of glucose to stimulate the rates of insulin release. There was thus a diminished insulin release in response to 16.5 mM glucose after infection of islets with either V89-4557 or PV-1 ($P < 0.05$ *vs.* controls). The insulin release in islets infected with the JVB strain, cultured at low glucose, was significantly higher than the rate of the controls indicating that a leakage of insulin could be detected as early as 72 h pi. The increase in the rate of insulin release after exposure of islets to 16.5 mM glucose as compared to 5.5 mM was 6 – 12 times in the control islets ($P < 0.05$ for all control groups). This increase was markedly diminished (to < 4 times) in islets infected with either V89-4557, JVB or PV-1. The low increase in the latter group reflected, however, an increased insulin release also in the infected islets cultured in parallel with 5.5 mM glucose.

In the last culture period (96 h pi), the rates of insulin release of islets cultured at 16.5 mM glucose, with one exception, returned to levels comparable to those shown in islets maintained at 5.5 mM glucose throughout the culture period. Only islets infected with the JVB strain showed a lasting high rate of insulin release also after return to a low-glucose (5.5 mM) culture medium. It is of note that islets infected with this strain also showed an increased rate of insulin release at 72 h pi at 5.5 mM glucose.

### DISCUSSION

The human islets used in the present study originated from pancreata excised from heart-beating donors, who differed both with regard to age, sex and genetic background. Variations between individual donors could therefore be expected, even if the islets, after being isolated, were maintained in culture media with uniform composition. Differences were indeed seen both with respect to virus multiplication in the islet cells, the CPE and the insulin response to glucose. Although the inherent heterogeneity of the islet cells justifies a cautious interpretation of the data, some conclusions can nevertheless be drawn.

First, the present observations indicate that each of the CBV-4 strains had a tropism but to various extent for the human islet cells since they all replicated in the human islets. Although in a few islet preparations, epithelial-like cell could be seen at the bottom of some wells after a few days of culturing, these cells never revealed any CPE and the virus titres were unaffected. This indicates that all viral replication took place in the islet cells. The question whether the ß-cells were infected with the VD2921 strain can not be clarified since there were no CPE and no effect on insulin release after culture in high glucose. However, the CBV-4 strains V89-4557 and JVB and PV-1 significantly affected the insulin release in some way, indicating that virus replication took place in the ß-cells. The different replication rate of the virus strains depended both on the strain itself and on the properties of the target donor cells, possibly indicating a disparity in the expression of cell surface molecules acting as virus receptors. The importance of the latter was particularly well illustrated by the $TCID_{50}$ titres of the V345 strain, which showed marked differences between different donors. To what extent this reflected individual differences between the expression of virus receptors on the islet cells, differences in islet cell viability or other factors remains to be established.

Second, all but one of the viruses caused CPE on the human islets ranging from mediocre (2+) to pronounced (4+) at seven days pi. The exception was islets infected with VD2921, which caused no CPE despite ongoing viral replication for up to 26 days pi. All the other strains caused at least some degree of lysis within the first four days. We therefore decided to carry out the measurements of insulin release from the infected islets cells within this time of culture in order to obtain results which reflected a true insulin response to a high glucose concentration rather than a mere leakage of insulin from lysed ß-cells. Our data provide a fair representation of functionally surviving insulin producing cells for 96 h pi when the islets were infected with the all of the CBV-4 strains except the JVB strain. A final culture in low glucose to as certain that the islets responded with a decrease in insulin release revealed that islets infected with all of the virus strains except JVB, returned to basal rates. Islets infected with the JVB strain continued to release significantly higher levels of insulin than the control islets, indicating that infection with this strain has in some way affected the ß-cells, resulting in a leakage of insulin. It has earlier been shown in mouse islets infected with an mouse pancreas-adapted strain of CBV-4 induced leakage of insulin when cultured in basal glucose concentrations.[26] It has been found that there is a selective "shut off" of the proinsulin synthesis by CBV-4.[27] In islets infected with V89-4557 and PV-1, almost all cells were lysed during a seven day period as shown by the trypanblue staining of the islets cells. This subtype of type! diabetes mellitus characterised by a rapid onset and an absence of diabetes-related antibodies has been reported by Imagawa *et al.* 2000.[28] Poliovirus serotype 1 (PV-1) belongs to the enterovirus genus and it has been associated with diabetes in a man following an infection,[29] it also showed tropism for the human islet cells resulting in lysis and decreased insulin response to glucose in this study. However, epidemiological data linking polio to IDDM are scarce and still insufficient to exclude a positive association between these two conditions.

In conclusion, all the virus strains used could replicate in human islet cells. To our knowledge this is the first time it has been shown that different strains of a serotype of Coxsackie B can differ in their replication pattern in human islet cells. The outcome of the infection differed. Infection with three of the strains, two CBV-4 strains and PV-1, affected the insulin release significantly compared with controls, indicating replication of virus in the ß-cells. One of the CBV-4 strains that was able to replicate in islet cells without causing CPE might *in vivo* initiate an autoimmune response due to its persistence in the islets cells. Persistently infected cells may show injured cell metabolism even if the infection is not lethal. When only a part of the islet cells is killed immediately and the functional capacity of the remaining cells is almost unaffected, an autoimmunity, perhaps by exposing intracellular proteins, might initiate the process leading to IDDM, *e.g.*, the strains that only caused mediocre CPE might also be diabetogenic.

### *Acknowledgements*

The authors are grateful to Dr. Decio Eizirik and Dr. Claes Hellerström for advice and encouragement during the course of this work. We are also grateful for the technical assistance of Mrs. Marianne Ericsson, to Dr. Tapani Hovi, National Public Health Institute, Helsinki, Finland, for kindly providing the CBV-4-E2 strain. The experiments, were supported by grants from the European Community (BMH4-CT-95-1561, BMH4-CT98-3952), the Swedish Diabetes Association, Gillbergska foundation, Novo Nordisk foundation and the Medical Research Council (K97-12XC-12445-01A, K98-12XC-12445-02B).

### *References*

[1] Szopa, T. M., Titchener, P. A., Portwood, N. D. and Taylor, K. W. (1993). Diabetes mellitus due to viruses – some recent developments. *Diabetologia*, **36**, 687–695.

[2] Yoon, J. W., Environmental factors in the pathogenesis of insulin-dependent diabetes mellitus. In: Pickup, J. C. and Williams, G. (Eds.), *Textbook of diabetes mellitus*. Blackwell Science Ltd., London, 1997, pp. 14.1–14.14.

[3] Ginsberg-Fellner, F., Witt, M. E. and Yagihaski, S. (1984). Congenital rubella-syndrome as a model for type 1 (insulin-dependent) diabetes mellitus: Increased prevalence of islet cell surface antibodies. *Diabetologia*, **27**, 87–89.

[4] Gamble, D. R. (1980). Relation of antecedent illness to development of diabetes in children. *British Medical Journal*, **2**, 99–101.

[5] Hyöty, H., Hiltunen, M., Knip, M., Laakonnen Vähäsalo, M., Karjalainen, J., Koskela, P., Roivainen, M., Leinikki, P., Hovi, T. and Åkerblom, H. K. (1995). Childhood Diabetes in Finland Study Group. A prospective study of the role of Coxsackie B and other enterovirus infections in the pathogenesis of IDDM. *Diabetes*, **44**, 652–657.

[6] King, M. L., Shaikh, A., Bidwell, D., Voller, A. and Banatvala, J. E. (1985). Coxsackie-B-specific IgM responses in children with insulin-dependent (juvenile-onset; type 1) diabetes mellitus. *Lancet*, **1**, 1397–1399.

[7] Frisk, G., Fohlman, J., Kobbah, M., Ewald, U., Tuvemo, T., Diderholm, H. and Friman, G. (1985). High frequency of Coxsackie-B-virus-specific IgM in children developing Type I diabetes during a period of high diabetes morbidity. *Journal of Medical Virology*, **17**, 219–227.

[8] Frisk, G., Nilsson, E., Tuvemo, T., Friman, G. and Diderholm, H. (1992). The possible role of Coxsackie A and Echo viruses in the pathogenesis of Type 1 diabetes mellitus studied by IgM analysis. *Journal of Infectious Diseases*, **24**, 13–22.

[9] Frisk, G. and Diderholm, H. (1997). Antibody responses to different strains of Coxsackievirus B4 in children with newly-diagnosed Type I diabetes mellitus. *Journal of Infection*, **34**, 205–210.

[10] Clements, G. B., Galbraith, D. N. and Taylor, K. W. (1995). Coxsackie B virus infection and onset of childhood diabetes. *Lancet*, **346**, 221–223.

[11] Andréoletti, L., Hober, D., Hober-Vandenberghe, C., Belaich, S., Vantyghem, M. C., Lefebvre, J. and Wattré, P. (1997). Detection of Coxsackie B virus RNA sequences in whole blood samples from adult patients at the onset of Type 1 diabetes mellitus. *Journal of Medical Virology*, **52**, 121–127.

[12] Yoon, J.-W., Austin, M., Onodera, T. and Notkins, A. L. (1979). Isolation of a virus from the pancreas of a child with diabetic ketoacidosis. *New England Journal Medicine*, **300**, 1173–1179.

[13] Champsaur, H. F., Bottazzo, G. F., Bertrams, J., Assan, R. and Bach, C. (1982). Virologic, immunologic, and gentic factors in insulin-dependent diabetes mellitus. *Journal of Pediatrics*, **100**, 15–20.

[14] Yoon, J. W., Viruses as triggering agents of insulin-dependent diabetes mellitus. In: Leslie RDG (Ed.), *The Causes of Diabetes*, London: John Whiley & Sons, 1993, pp. 83–103.

[15] Hou, J., Said, C., Franchi, D., Dockstader, P. and Chatterjee, N. K. (1994). Antibodies to glutamic acid decarboxylate and P2-C peptides in sera from Coxsackie-virus B-4 infected mice and IDDM patients. *Diabetes*, **43**, 1260–1266.

[16] Lönnrot, M., Hyöty, H., Knip, M., Roivainen, M., Kulmala, P., Leinikki, P. and Åkerblom, H. K. (1996). Childhood Diabetes in Finland Study Group. Antibody cross-reactivity induced by the homologous regions in glutamic acid decarboxylase (GAD65) and 2C protein of Coxsackievirus B4. *Clinical Experimental Immunology*, **104**, 389–405.

[17] Conrad, B., Weissmahr, R. N., Böni, J. and Mach, B. (1997). A human endogenous retroviral superantigen as candidate autoimmune gene in Type 1 diabetes. *Cell*, **90**, 303–313.

[18] Kim, A., Jun, H. S., Wong, L., Stephure, D., Pacaud, D., Trussell, R. A. and Yoon, J. W. (1999). Human endogenous retrovirus with a high genomic sequence homology with IDDMK (1,2) is not specific for Type I (insulin-dependent) diabetic patients but ubiquitous. *Diabetologia*, **42**, 413–418.

[19] Horwitz, M. S., Bradley, L. M., Harbertson, J., Krahl, T., Lee, J. and Sarvetnick, N. (1998). Diabetes induced by Coxsackie virus: Initiation by bystander damage and not molecular mimicry. *Nature Medicine*, **4**, 781–785.

[20] Eizirik, D. L., Pipeleers, D. G., Ling, Z., Welsh, N., Hellerström, C. and Andersson, A., Major species differences between humans and rodents in the susceptibility to pancreatic-cell injury. *Proceedings of National Academic Science*, 1994, **91**, 9253–56.

[21] Titchener, P. A., Genetic studies on Coxsackie B4 tissue tropism. *Ph.D. Thesis*, University of Reading, 1991 Reading UK.

[22] Hartig, P. C., Madge, G. E. and Webb, S. R. (1983). Diversity within a human-isolate of Coxsackie B4: relationship to viral-induced diabetes. *Journal of Medical Virology*, **11**, 23–30.

[23] Keymeulen, B., Ling, Z., Gorus, F. K., Delvaux, G., Bouwens, L., Grupping, A., Hendrieck, C., Pipeleers-Marichal, M., Van Schravendijk, C., Salmela, K. and Pipeleers, D. G. (1998). Implantation of standardized beta-cell grafts in liver segment of IDDM patients:graft and recipients characteristics in two cases of insulin-independence under maintenance immunosuppression for prior kidney graft. *Diabetologia*, **41**, 452–459.

[24] Eizirik, D. L., Korbutt, G. S. and Hellerström, C. (1992). Prolonged exposure of human pancreatic islets to high glucose concentrations *in vitro* impairs the beta-cell function. *Journal of Clinical Investigation*, **90**, 1263–1268.

[25] Yoon, J. W., Ausin, M., Onodera, T. and Norkins, A. L. (1979). Isolation of a virus from the pancreas of a child with diabetic ketoacidosis. *New England Journal of Medicine*, **24**, 1173–1179.

[26] Szopa, T. M., Gamble, D. R. and Taylor, W. (1986). Coxsackie B4 virus induces short-term changes in the metabolic functions of mouse pancreatic islets *in vitro*. *Cell Biochemistry and Function*, **4**, 181–187.

[27] Poortwood, N. D. and Taylor, K. W. (1990). Coxsackie B4 virus-induced changes in mouse pancreatic ß-cell m-RNAs. *Biochm. Soc. Trans.*, **18**, 1264.

[28] Inagawa, A., Hanafusa, T., Miyagawa, J. and Matsuzawa, Y. (2000). A novel subtype of Type 1 diabetes mellitus characterized by a rapid noset and an absence of diabetes-related antibodies. *New England Journal of Medicine*, **342**, 301–307.

[29] John, H. J. (1934). The diabetic child. Ethiologic factors. *Ann. Internal Medicine*, **8**, 198–213.

